# Social correlates of cigarette smoking among Icelandic adolescents: A population-based cross-sectional study

**DOI:** 10.1186/1471-2458-8-86

**Published:** 2008-03-07

**Authors:** Alfgeir L Kristjansson, Inga D Sigfusdottir, John P Allegrante, Asgeir R Helgason

**Affiliations:** 1 Centre for Social Research and Analysis, School of Health and Education, Reykjavik University, Reykjavik, Iceland; 2 Department of Oncology-Pathology, Karolinska Institute, Stockholm, Sweden; 3 Department of Health and Behavior Studies, Teachers College, and Department of Sociomedical Sciences, Mailman School of Public Health, Columbia University, New York, USA; 4 Centre of Public Health, Stockholm, Sweden

## Abstract

**Background:**

Previous research has shown that between 80 and 90 percent of adult smokers report having started smoking before 18 years of age. Several studies have revealed that multiple social factors influence the likelihood of smoking during adolescence, the period during which the onset of smoking usually occurs. To better understand the social mechanisms that influence adolescent smoking, we analyzed the relationship and relative importance of a broad spectrum of social variables in adolescent smoking in Iceland, a Nordic country with high per-capita income.

**Methods:**

We used cross-sectional data from 7,430 14- to 16 year-old students (approximately 81% of all Icelanders in these age cohorts) in the 2006 *Youth in Iceland *study. The *Youth in Iceland *studies are designed to investigate the role of several cognitive, behavioral, and social factors in the lives of adolescents, and the data collected are used to inform the design, implementation, and evaluation of substance use prevention programs that are being developed by Icelandic social scientists, policy makers, and practitioners.

**Results:**

Our analysis revealed that friends' smoking behavior and attitude toward smoking were strongly associated with adolescent smoking and other tobacco use, as well as alcohol consumption during the previous 30 days. Main protective factors were parent's perceived attitude toward smoking, the quantity of time spent with parents, absence of serious verbal conflict between parents and adolescents, and participation in physical activity. Family structure was related to adolescent smoking to a small extent, but other background factors were not.

**Conclusion:**

We conclude that multiple social factors are related to adolescent smoking. Parents and other primary preventive agents need to be informed about the complicated nature of the adolescent social world in order to maximize their impact.

## Background

Cigarette smoking is one of the major causes of morbidity and premature death in countries with advanced economies [1,2]. Research among adult smokers reveals that between 80 and 90% of them began to smoke before 18 years of age [3,4]. Understanding the social context of adolescent smoking is an important step in designing effective smoking prevention efforts. This has recently been emphasized in writings discussing epidemiological methods to assess risk factors for health risk behaviors such as smoking in adolescence [5-7].

Several studies have revealed that multiple social factors influence the likelihood of smoking during adolescence, the period during which the onset of smoking usually occurs [8-10]. These include family structure [11], socio-economic status [12], perceived parental expectations [13], parental support [8], parental monitoring [13], parental practices [14], parental smoking behavior [2], sibling smoking behavior [15], peer smoking [4], perceived acceptance of friends [13], use of other legal and illegal substances [16], and participation in physical activity [17]. Many of these factors have furthermore been illuminated in previous Icelandic studies [8,17]. Recently, researchers have called for a more comprehensive modeling of adolescent smoking behavior by including more variables in analyses in order to more clearly understand the complex nature of the social influences on adolescent smoking [18].

In the present study, we analyzed the relationship and relative importance of a broad spectrum of social variables in adolescent smoking in Iceland, a Nordic country with high per-capita income. We included in our analyses several background factors, relationship with parents, family conflict, smoking of significant others, and personal attributes such as participation in physical activity, academic achievement, and substance use. Thus, the aim of our analysis was to investigate how multiple social factors are related to adolescent cigarette smoking, to assess important social features that increase or decrease the likelihood of adolescent smoking, and to demonstrate the importance of the complex nexus of adolescent smoking.

## Methods

### Sample

The data for this study came from the 2006 Icelandic study, *Youth in Iceland*. The sample includes all students aged 14 to 16 years who were enrolled in the 9th (14- and 15-years-olds) and 10th (15- and 16-year-olds) grades in all Icelandic secondary schools during March 2006. The study respondents in this sample represent approximately 81% of the national population of Iceland in these age groups. All aspects of the data collection were supervised by the Icelandic Centre for Social Research and Analysis (ICSRA) at Reykjavik University. The Youth in Iceland studies are conducted with annual cross-sectional surveys in collaboration between the Ministry of Education, Science and Culture, the municipalities around the country of Iceland, and ICSRA. A passive consent is obtained from parents and all participants receive a covering letter 2–3 days prior to the survey taking place. The overall goal of these studies is to monitor various health behaviors of Icelandic youth, as well as risk and protective factors, with the overall purpose of improving the health status of children and young people. Various internationally validated measuring scales are used in the questionnaire, as well as components developed by ICSRA and its predecessor the Institute for Educational Research.

### Procedure

The Centre supervised the distribution of anonymous questionnaires and envelopes to all secondary schools in Iceland. Teachers at individual school sites assisted the students with their participation in the study and administered the survey questionnaire returns. All students who attended school on the day that the questionnaire was scheduled to be administered completed the questionnaire inside their classrooms. Students were instructed not to write their names or social security numbers, or any other identifying information, anywhere on the questionnaire. They were instructed to complete the entire questionnaire, but to ask for help if they had any problems or had any questions for clarification. Once students had completed the questionnaires, they were asked to place their completed questionnaire in the envelopes and seal it before returning the questionnaire to the supervising member of staff.

A total of 7430 students (51.4% girls) completed the questionnaire. An overwhelming majority of residents in Iceland (approximately 94%) are of Nordic and Anglo-Saxon descent, or both, and about 88% belongs to the Lutheran state Church [19]. This implies that differences in descent or religion in the Icelandic population should not serve to confound the study findings. The Icelandic school system is fundamentally based on schools run by the local county councils under the administration of the ministry of education and most of them use the same curriculum.

### Measures

The level of smoking by the adolescents is captured with the question, "How much have you smoked on average during the last 30 days?" Response categories include: 1 = "Nothing," 2 = "Less than one cigarette per week," 3 = "Less than one cigarette per day," 4 = "1–5 cigarettes per day," 5 = "6–10 cigarettes per day," 6 = "11–20 cigarettes per day" and 7 = "more than 20 cigarettes per day." In our analyses we distinguish between those who do not smoke (response category 1), those who smoke occasionally (response categories 2 and 3), and those who smoke daily (response categories 5, 6, and 7). All variables used in the study are shown in Table 1, as are the proportions of respondents in each response category within each variable. The overall count of variables in the study, including items used in combined scaled responses, is 43. All measures in the study have been dichotomized in accordance to response categories shown in Table 1. We utilize a median-split approach, unless stated otherwise, in Table 1, in the coding of all continuous and scale variables in the study. Parental support, time spent with parents, parental control, and academic achievement were all constructed from discrete scales with 2 to 9 questions. In order to determine if multicollinearity might be a problem in our data we carried out an inter-item correlational analysis of the predictor variables. It revealed no dangerously high relationships.

**Table 1:** Population characteristics in study

**Characteristic**	**% (n/N)**
**Dependent variables**	

Never smokes	84.5% (6809/7203)
Smokes occasionally	6.7% (482/7203)
Smokes daily	8.8% (632/7203)

**Demographics**	

Gender	
Boys	49.9% (3612/7232)
Girls	50.1% (3620 (7232)
Family structure	
Lives with both parents	70.3% (5141/7309)
Lives under other arrangements	29.7% (2168/7309)
Mother's education	
College or higher	87.6% (6334/7227)
Secondary school or less	12.4% (893/7227)
Father's education	
College or higher	90.7% (6577/7254)
Secondary school or less	9.3% (677/7254)
Family income	
Better off	58.2% (4227/7263)
Worse off	41.8% (3036/7263)

**Relationship with parents**	

Parental support (5 items, CA = .86)	
More support	54.0% (3888/7195)
Less support	46.0% (3307/7195)
Time spent with parents (2 items, CA = .77)	
More time	41.2% (2988/7244)
Less time	58.8% (4256/7244)
Parental control (9 items, CA = .81)	
More control	44.0% (3120/7086)
Less control	56.0% (3966/7086)
Perceived parental reactions if one would smoke cigarettes	
Very much/much against	91.8% (6506/7090)
Rather against/do not care	8.2% (584/7090)

**Family conflict**	

Interparental serious verbal conflict	
Never	77.1% (5726/7426)
Yes, at some point	22.9% (1700/7426)
Parent-adolescent serious verbal conflict	
Never	63.2% (4695/7426)
Yes, at some point	36.8% (2731/7426)
Interparental violence	
Never	93.6% (6949/7426)
Yes, at some point	6.4% (477/7426)
Parent-adolescent violence	
Never	94.8% (7041/7426)
Yes, at some point	5.2% (385/7426)

**Smoking of significant others**	

Father's smoking	
No	71.2% (4373/6141)
Yes	28.8% (1768/6141)
Mother's smoking	
No	72.7% (4562/6277)
Yes	27.3% (1715/6277)
Sibling's smoking	
No	76.7% (4679/6099)
Yes	23.3% (1420/6099)

**Peer group**	

Perceived respect from peers if I would smoke cigarettes	
Less respect	93.5% (6831/7308)
More respect	6.5% (477/7308)
Friends cigarette smoking	
None	41.4% (2952/7132)
Some-most-all	58.6% (4180/7132)

**Participant social characteristics**	

Physical activity (3 items, CA = .68)	
More active	42.7% (2975/6962)
Less active	57.3% (3987/6962)
Healthy nutrition important	
Agrees	87.9% (6119/6959)
Disagrees	12.1% (840/6959)
Academic Achievement (4 items, .80)	
Better half	49.8% (3490/7007)
Poorer half	50.2% (3517/7007)
Alcohol consumption during last 30 days (Any consumption)	
No	66.3% (4724/7128)
Yes	33.7% (2404/7128)
Smoke free tobacco use during last 30 days (chewing tobacco or snuff)	
No	86.9% (6240/7181)
Yes	13.1% (941/7181)

### Statistical analyses

We first calculated crude odds ratios (OR) to evaluate the risk of exposure effects in the group under study relative to the reference group and associated 95% confidence intervals [20]. We then used multivariate logistic regression models with OR to adjust for the possible confounding influences between the independent variables on the dependent one in each model [21].

## Results

Of all student included in the study base, essentially the entire population of Icelandic 9^th ^and 10^th ^graders, 81% responded to the questionnaire. A validity check on the remaining 19% of the population that did not participate in the study (did not attend schools this particular day) revealed no particular difference between non-participants and participants. Population characteristics of the study sample are presented in Table 1. For discrete responses the number of items are shown and the relevant Cronbach's Alpha (CA) calculation of internal consistency.

Table 2 shows the unadjusted and adjusted Odds Ratio calculations with 95% confidence intervals for adolescents that smoke either occasionally or daily. The variables expected to be protective against tobacco use are treated as the reference group. The demographic variables for parental education and family income were not related to the likelihood of adolescent smoking in the adjusted analysis but children not living with both parents had an increased risk of being daily smokers (OR = 1.52, 95% CI = 1.09–2.13). Parental support and parental control were significantly related to daily smoking in the crude analysis but not in the adjusted analysis. However, "time spent with parents" was significant in both the crude and adjusted analyses for daily smoking (adjusted OR = 2.27, 95% CI = 1.50–3.44) but not occasional smoking.

**Table 2:** Unadjusted and adjusted odds ratios (OR) with 95% confidence intervals (CI) for adolescent daily cigarette smoking and occasional smoking

**Variables**	**Occasional Smoking**			**Daily Smoking**		
		
	**% n/N**^a^	**Unadjusted OR (95% CI)**	**Adjusted OR (95% CI)**^b^	**% n/N**^a^	**Unadjusted OR (95% CI)**	**Adjusted OR (95% CI)**^b^
Family structure						
Both parents	6.7% (314/4700)	1.0		6.6% (309/4695)	1.0	
Other arrangements	9.3% (167/1793)	1.44 (1.18–1.75)	0.99 (0.73–1.36)	16.3% (317/1943)	2.76 (2.34–3.27)	1.52 (1.09–2.13)
Mother's education						
College or higher	7.1% (401/5687)	1.0		8.5% (490/5776)	1.0	
Secondary school or less	10.0% (74/741)	1.46 (1.13–1.90)	1.06 (0.70–1.59)	16.1% (128/795)	2.07 (1.68–2.56)	1.01 (0.66–1.55)
Father's education						
College or higher	7.2% (425/5892)	1.0		8.8% (529/5996)	1.0	
Secondary school or less	8.4% (47/561)	1.18 (0.86–1.61)	0.90 (0.54–1.48)	15.3% (93/607)	1.87 (1.47–2.37)	1.05 (0.64–1.73)
Family income						
Better off	7.2% (268/3723)	1.0		10.5% (406/3861)	1.0	
Worse off	7.4% (204/2743)	1.04 (0.86–1.25)	1.07 (0.80–1.44)	7.8% (215/2754)	0.72 (0.61–0.86)	0.97 (0.68–1.36)
Parental support						
More	6.2% (220/3573)	1.0		6.6% (237/3590)	1.0	
Less	8.6% (245/2857)	1.43 (1.18–1.73)	0.80 (0.59–1.07)	12.5% (373/2985)	2.02 (1.70–2.40)	0.90 (0.65–1.26)
Time spent with parents						
More	4.3% (122/2836)	1.0		3.2% (91/2805)	1.0	
Less	9.6% (347//3614)	2.36 (1.91–2.92)	1.33 (0.97–1.84)	13.8% (525/3792)	4.79 (3.81–6.02)	2.27 (1.50–3.44)
Parental control						
More	5.4% (157/2909)	1.0		5.5% (159/2911)	1.0	
Less	8.8% (302/3441)	1.67 (1.38–2.06)	0.91 (0.68–1.23)	12.3% (439/3578)	2.42 (2.01–2.92)	1.12 (0.79–1.59)
Perceived parental reactions if one would smoke cigarettes						
Very much/much against	6.7% (409/6126)	1.0		5.4% (325/6042)	1.0	
Rather against/do not care	20.7% (61/294)	3.66 (2.71–4.94)	2.35 (1.49–3.72)	54.8% (282/515)	21.29 (17.32–26.17)	8.92 (6.04–13.17)
Interparental serious verbal conflict						
No	6.1% (314/5148)	1.0		7.5% (392/5226)	1.0	
Yes, at some point	11.8% (168/1423)	2.06 (1.69–2.51)	1.19 (0.86–1.64)	16.1% (240/1495)	2.36 (1.99–2.80)	1.04 (0.72–1.50)
Parent-adolescent serious verbal conflict						
No	4.4% (190/4334)	1.0		4.6% (200/4344)	1.0	
Yes, at some point	13.1% (292/2237)	3.27 (2.71–3.96)	1.72 (1.27–2.33)	18.2% (432/2377)	4.60 (3.86–5.49)	2.83 (1.98–4.04)
Interparental violence						
No	6.7% (419/6224)	1.0		8.2% (516/6321)	1.0	
Yes, at some point	18.2% (63/347)	3.07 (2.30–4.11)	1.74 (1.02–2.96)	29.0% (116/400)	4.60 (3.64–5.81)	0.80 (0.44–1.47)
Parent-adolescent violence						
No	6.9% (434/6312)	1.0		8.2% (522/6400)	1.0	
Yes, at some point	18.5% (48/259)	3.08 (2.22–4.28)	0.86 (0.46–1.60)	34.3% (110/321)	5.87 (4.58–7.52)	1.53 (0.85–2.78)
Father's smoking						
No	6.4% (259/4061)	1.0		7.0% (287/4089)	1.0	
Yes	9.4% (139/1483)	1.52 (1.22–1.88)	1.03 (0.74–1.42)	17.1% (277/1621)	2.73 (2.29–3.26)	1.12 (0.80–1.59)
Mother's smoking						
No	6.6% (282/4261)	1.0		6.5% (276/4255)	1.0	
Yes	8.9% (126/1411)	1.38 (1.11–1.72)	1.04 (0.74–1.47)	18.5% (292/1577)	3.28 (2.75–3.91)	1.69 (1.20–2.40)
Sibling's smoking						
No	6.1% (268/4366)	1.0		6.6% (289/4387)	1.0	
Yes	11.5% (132/1150)	1.98 (1.59–2.47)	1.19 (0.87–1.65)	20.3% (260/1278)	3.62 (3.02–4.34)	1.55 (1.10–2.18)
Perceived respect from peers if would smoke cigarettes						
Less respect	6.5% (403/6223)	1.0		7.2% (452/6272)	1.0	
More respect	24.6% (72/293)	4.71 (3.54–6.25)	2.25 (1.43–3.53)	43.6% (171/392)	9.96 (7.98–12.43)	3.27 (2.09–5.13)
Friends cigarette smoking						
None	1.2% (34/2910)	1.0		0.5% (14/2890)	1.0	
Some-all	12.5% (443/3547)	12.07 (8.49–17.17)	5.68 (3.38–9.55)	16.1% (595/3699)	39.38 (23.12–67.06)	17.17 (6.18–47.71)
Physical activity						
More	4.8% (135/2839)	1.0		3.9% (110/2814)	1.0	
Less	9.3% (322/3481)	2.04 (1.66–2.51)	1.33 (0.98–1.80)	13.1% (476/3635)	3.70 (2.99–4.59)	2.15 (1.48–3.13)
Healthy nutrition important						
Agree	6.9% (389/5628)	1.0		7.7% (435/5674)	1.0	
Disagree	10.1% (69/684)	1.51 (1.15–1.98)	0.94 (0.62–1.42)	19.5% (149/764)	2.92 (2.38–3.58)	1.35 (0.89–2.03)
Academic Achievement						
Higher	5.1% (169/3330)	1.0		3.6% (119/3280)	1.0	
Lower	9.8% (288/2929)	2.04 (1.68–2.48)	1.17 (0.88–1.57)	14.9% (463/3104)	4.66 (3.78–5.73)	2.12 (1.49–3.02)
Alcohol consumption 30 days						
No	2.0% (92/4635)	1.0		1.4% (65/4608)	1.0	
Yes	21.1% (384/1816)	13.24 (10.47–16.75)	8.81 (6.13–12.64)	28.1% (560/1992)	27.33 (21.00–35.58)	7.64 (5.11–11.41)
Smoke free tobacco use						
No	4.9% (290/5882)	1.0		5.6% (330/5922)	1.0	
Yes	29.4% (185/630)	8.02 (6.51–9.87)	3.57 (2.56–4.98)	39.9% (296/741)	11.27 (9.38–13.55)	5.74 (3.93–8.37)

^a ^Denominators vary from N as a result of differences in missing information.

^b ^The relationship with each variable in this column is adjusted for all other variables in the analysis and gender.

The family conflict variables, "interparental serious verbal conflict," and "parent-adolescent violence" were only related to smoking in the crude analysis but not in the adjusted models for both daily and occasional smoking but "interparental violence" was borderline significant in the adjusted analyses for occasional smoking. However, the "parent-adolescent serious verbal conflict" variable was significant in the crude- and adjusted analysis for both daily smoking (adjusted OR = 2.83, 95% CI = 1.98–4.04) and occasional smoking (adjusted OR = 1.72, 95% CI = 1.27–2.33). In addition, father's, mother's, and sibling's smoking all increase the risk of adolescent daily smoking in the crude analysis but father's smoking was not significant in the adjusted analysis whereas mother's and sibling's smoking continues to be of importance. Of all the family factors in the study "perceived parental reactions if one would smoke" is the most important risk factor both in the crude and adjusted analyses (adjusted OR for daily smoking = 8.92, 95% CI = 6.04–13.17). Expecting more respect from peers if one would smoke increases the risk in the adjusted analysis for daily smoking (OR = 3.27, 95% CI = 2.09–5.13), and occasional smoking (OR = 2.25, 95% CI = 1.43–3.53).

Moreover, the risk of being a daily smoker increases to over 17-fold when peers smoke when adjusted for other factors in the analysis (OR = 17.17, 95% CI = 6.18–47.71). This is indeed the strongest risk factor for daily smoking in the study. Finding healthy nutrition important was not a significant factor for daily smoking after adjustment. To the contrary, engaging in more physical activity (adjusted OR for daily smoking = 2.15, 95% CI = 1.48–3.13) and achieving better in school (adjusted OR for daily smoking = 2.12, 95% CI = 1.49–3.02) were negatively related to smoking. However, academic achievement was not significantly related to occasional smoking in the adjusted analysis. Drinking alcohol during last 30 days was strongly related to daily smoking (OR = 7.64, 95% CI = 5.11–11.41) and occasional smoking (OR = 8.81, 95% CI = 6.13–12.64), and smoke-free tobacco use was strongly related to both daily (OR = 5.74, 95% CI = 3.93–8.37) and occasional smoking (OR = 3.57, 95% CI = 2.56–4.98) after adjustment.

## Discussion

We found that the smoking behavior of friends and their attitude toward smoking were social factors strongly associated with adolescent cigarette smoking. Another strong force protecting adolescents from smoking was parents attitudes towards smoking, the quantity of time spent with parents, and absence of "serious" verbal conflict between parents and child. Alcohol consumption during the previous 30 days greatly increased the risk of smoking both in the unadjusted and adjusted analysis for both daily and occasional smoking.

In this study demographic factors seem to be of little importance for adolescent smoking, probably reflecting the homogeneity of the Icelandic population and relatively well developed social security system. Parental support, parental control and time spent with parents are all protective factors of adolescent smoking in the crude analysis but only time spent with parents continues to be of importance in the adjustment models. This is consistent with prior knowledge from Iceland and is presently one of the cornerstones in the Icelandic Model of substance use prevention (a detailed paper describing this prevention approach is currently in submission). Moreover, this study supports the notion that the quantity of time parents spend with their children may be a major protective factor for adolescent smoking. But the adolescents' perception that parents are not against adolescent smoking greatly increases the risk of being a daily smoker, independent of other factors (OR = 8.92, CI = 6.04–13.17). However, since this study was carried out with a cross-sectional design, this finding may reflect that parents are perceived to tolerate the smoking of adolescents rather than being a risk factor for smoking initiation. Yet, data from other studies indicate that parental expectations do function as a protective factor for the initiation of adolescent smoking [13], which is consistent with our preferred interpretation of these findings.

Family conflict has been reported to be strongly related with increased risk of adolescent smoking [22,23]. The social context of this relationship is not fully understood. Findings in this study indicate that it may particularly be serious verbal conflicts between the adolescent and his/her parents that is of importance, being the only family-conflict variable of the four (with interparental serious verbal arguments, interparental violence, and parent-adolescent violence) that remains significantly related to daily smoking after adjustments for other variables in the analysis. This issue will need further study in the future.

Although mothers' and sibling smoking remained significantly related to adolescent smoking after adjustments, and this relationship has shown to be of importance in previous studies [24], the impact was relatively small in comparison to other factors with adjusted odds ratio of 1.69 (CI = 1.20–2.40) for mother's smoking and 1.55 (CI = 1.10–2.18) for sibling's smoking. Reporting having friends that smoke increased the risk of being a daily smoker 17-fold when adjusted for other variables in the analysis. This was by far the strongest risk factor in the study after adjusting for other factors. The reason for this high odds ratio is the group division of our dependent variable. Most public health studies on adolescent smoking divide participants into "never" and "ever" smokers. In this study, those who smoke daily are distinguished from those who smoke occasionally and both groups are analyzed against those who do not smoke at all. The present finding nevertheless supports previous research that the smoking of peers is of central importance for adolescent smoking [8,17,25]. Recently, however, this notion has been doubted by Arnett [26] who argues that we need to look more closely into the peer-selection process to fully understand the important role of peer smoking in adolescent smoking.

One problem with interpreting the role of peer influences is that we have no data on the order of initiation to smoking in the peer group and consequently do not know who influenced whom. Notwithstanding, what we can safely say is that birds of a feather tend to flock together when it comes to adolescent tobacco smoking. Also, by including in the model a measure of perceived respect from peers, if one would smoke cigarettes, in addition to the smoking of peers, we may be able to analyze further the influence of the peer group. In this study, if smoking cigarettes was perceived to increase peer respect, the odds of daily smoking was 3.27 in the adjusted analyses, down from an OR = 9.96 in the crude analysis which is probably because of other protective factors in the model.

Finally, higher levels of physical activity and academic achievement were protective factors to the risk of adolescent daily smoking after adjustment. This is consistent with the findings of previous studies [8]. In contrast, alcohol consumption during the last 30 days and having used smoke-free tobacco greatly increase the risk of daily and occasional smoking, underlining the fact that it is often the same individuals who engage in risk behaviors of more than one kind.

Due to the study's cross-sectional design we were not able to draw any firm conclusions regarding causality between the dependent and independent variables. Also, small adjusted odds ratio values in a sample of this size (7,430 respondents) should be interpreted with caution, particularly when the 95% confidence intervals are close to, but do not include, 1.0 [27].

## Conclusion

The present findings indicate that multiple social factors are related to adolescent smoking. Important factors protecting adolescents from smoking included higher academic achievement and physical activity. Abstinence from other tobacco products and alcohol were also protective factors; however, the strongest protective factors comprised peers abstinence from smoking and their perceived negative attitudes to smoking. Also, parent's negative attitudes to smoking, absence of serious verbal arguments with parents and the quantity of time spent with parents, were highly protective against adolescents smoking in this study.

## Competing interests

The author(s) declare that they have no competing interests.

## Authors' contributions

ÁLK conceptualized the study, performed most of the data analyses, and contributed substantially to the writing and referencing. IDS procured funding to support the original data collection and analysis and edited multiple drafts of the manuscript. JPA contributed substantially to the writing and referencing and edited multiple drafts of the manuscript. ÁRH was the principal editor and contributed substantially to the writing and referencing. All authors have read and approved the final manuscript.

## Pre-publication history

The pre-publication history for this paper can be accessed here:


